# The Effect of Renalase rs2576178 and rs10887800 Polymorphisms on Ischemic Stroke Susceptibility in Young Patients (<50 Years): A Case-Control Study and In Silico Analysis

**DOI:** 10.1155/2021/5542292

**Published:** 2021-09-22

**Authors:** Nourollah Ramroodi, Azar Khorrami, Seyed Mehdi Hashemi, Mahnaz Rezaei, Hossein Shahraki-ghadim, Saeedeh Salimi

**Affiliations:** ^1^Department of Neurology, School of Medicine, Zahedan University of Medical Sciences, Zahedan 9816743111, Iran; ^2^School of Medicine, Zahedan University of Medical Sciences, Zahedan 9816743111, Iran; ^3^Clinical Immunology Research Center, Ali-Ebne Abitaleb Hospital, Zahedan University of Medical Sciences, Zahedan 9816743111, Iran; ^4^Department of Internal Medicine, School of Medicine, Zahedan University of Medical Sciences, Zahedan 9816743111, Iran; ^5^Department of Clinical Biochemistry, School of Medicine, Zahedan University of Medical Sciences, Zahedan 9816743175, Iran; ^6^Cellular and Molecular Research Center, Resistant Tuberculosis Institute, Zahedan University of Medical Sciences, Zahedan 9816743111, Iran

## Abstract

**Background:**

Ischemic stroke (IS) is the most common form of cerebrovascular accident which its precise etiology remains mysterious. Renalase is a catecholamine-degrading enzyme playing a major role in blood pressure control. Recent studies show the effect of renalase activity on various diseases like IS. In the current study, we examined the possible effects of renalase gene (*RNLS*) rs2576178 and rs10887800 variants at the 5′-flanking and intron 6 regions on IS, respectively.

**Methods:**

One hundred and fifty-four IS patients younger than 50 years and 165 age- and sex-matched controls were recruited in the study. For genotyping of rs2576178 and rs10887800 variants, the PCR-RFLP method was used.

**Results:**

The *RNLS* rs10887800 AG genotype was more repeated in IS patients, but the difference was marginally nonsignificant (*P* = 0.054). This variant was associated with IS in the overdominant model, and the AG genotype is associated with a1.6-fold increased risk of IS compared to AA+ GG genotypes (OR = 1.6, 95% CI: 1-2.5, *P* = 0.033). No relationship was observed between *RNLS* rs2576178 polymorphism and IS in all genetic models. The findings of the haplotype and combination effects of rs10887800 and rs2576178 variants on IS showed no significant association. The in silico analysis showed no effect of rs2576178 and rs10887800 polymorphisms in the RNA structure, but the alteration of RNA sequence in rs2576178 results in the lack of a MBNL1 protein binding site.

**Conclusions:**

*RNLS* rs10887800 but not rs2576178 polymorphism was associated with IS susceptibility in the overdominant model (AG vs AA+ GG genotypes).

## 1. Introduction

Stroke is a cerebrovascular accident, identified as one of the leading causes of morbidity and mortality worldwide. It is the third important reason of death after cardiovascular diseases and cancer, responsible for about %10 of all deaths [1]. Evidences show that seventy percent of strokes and eighty seven percent of stroke-related deaths are occurred in low- and middle-income countries, and the prevalence of stroke has elevated in these regions over the past four decades [2]. In addition, the mean age showed a 15-year decrease in stroke patients, and the death rate is elevated in these regions [3]. According to the World Health Organization (WHO), it is the second leading cause of death in Iran [4] . Commonly stroke occurs in productive age and is mostly preventable and treatable. There are two common types of stroke including brain ischemia as result of thrombosis, embolism, systemic hypoperfusion or blood disorders, and brain hemorrhage [5].

Ischemic stroke (IS) is the most common form of this complication (more than 80%), initiated from obstruction of one or more cerebral arteries and subsequently a serious decrease of local cerebral blood flow. This complication results in inadequate glucose and oxygen levels which is necessary for brain cells. Then, the ischemia promotes a series of cellular and molecular responses which can lead to brain damage and focal inflammation [6, 7].

The precise etiology of IS is not yet fully understood; although, several risk factors including age, hypertension, and diabetes mellitus as well as genetic and environmental factors may contribute to its pathogenesis. In addition, the role of genetic variants in IS susceptibility is predictable similar to other vascular complications [8].

Renalase is a FAD-dependent amine oxidase enzyme expressed in various cells; however, its expression is higher in the kidney, heart, skeletal muscle, and liver. This enzyme is involved in the catecholamine degradation; therefore, it plays a significant role in the regulation of blood pressure and cardiac function [9]. Moreover, renalase seems to function as a cytokine that is completely independent of its enzymatic activity [10]. Evidences showed that the basal activity of renalase is very low; however, after catecholamine administration or increased blood pressure, elevated activity of the enzyme lead to a reduction in blood pressure [11]. In addition, a study conducted on recombinant renalase showed that it decreases blood pressure in vivo via reducing cardiac output and peripheral vascular tone [12].

Several studies demonstrated the association of renalase with hypertension and ischemic-related diseases [13]. The effect of renalase on stroke was first investigated in a study conducted on renalase variants and type 2 diabetes [14, 15]. The role of renalase has been confirmed by lower serum renalase in patients on hemodialysis with a history of stroke than in those without stroke [15]. Therefore, several studies were performed on the effects of renalase gene (RNLS) polymorphisms on several diseases including hypertension, preeclampsia, CAD, and IS [14, 16].

The RNLS gene is located on chromosome10 (10q23.31) [9]. There are several polymorphisms in the RNLS gene which their impacts on different disorders have been considered. Among them, a variant at intron 6 close to the exon/intron boundary (rs10887800) and another one at the 5′-flanking region (rs2576178) are more studied [14]. Previous studies showed the association between rs10887800 and rs2576178 variants and ischemic stroke in Poland and China [14, 16, 17], however; there is no study in other countries.

Regarding the role of renalase in hypertension and ischemic-related diseases and the being rare published report about the effects of renalase polymorphisms on stroke; in the current study, we investigated the probable effects of *RNLS* rs2576178 and rs10887800 polymorphisms on IS in young patients.

## 2. Materials and Methods

### 2.1. Study Participants

One hundred and fifty-four IS patients younger than 50 years and 165 age- and sex-matched controls were recruited from Ali-Ebne Abitaleb Hospital of Zahedan University of Medical Sciences between June 2018 and February 2020. The IS patients were selected from hospital-based patients whose diseases were recognized according to clinical symptoms, computerized tomography(CT), or magnetic resonance imaging (MRI) scan by a neurologist. The study protocol was approved by the ethical committee and also, the participants signed a written informed consent.

Demographic and clinical characteristics and lifestyle of both groups were obtained by the skilled nurses. The subjects who used tobacco regularly during 6 months ago (at least one cigarette per week) were known as smoker.

### 2.2. Genomic DNA Extraction and Genotyping

The salting out method was used to separate genomic DNA from peripheral blood leukocytes. To genotype *RNLS* variants, PCR-RFLP was done as described previously [18, 19].

### 2.3. Statistical Analysis

SPSS version 20 (SPSS Inc., Chicago, IL, USA) was used to examine the data. The comparison of clinical and demographic data was done by the Fisher exact or Student *t*-tests. To analyze the effect of each genotype on disease, logistic regression analyses were performed based on OR and 95% CI. HaploView software was employed to examine the frequency and LD of haplotypes. *P* < 0.05 was considered statistically significant. RNAsnp (https://rth.dk/resources/rnasnp/) and SpliceAid2 (http://193.206.120.249/splicing_tissue.html) were used for in silico analysis. RNAsnp predicts the structural changes in the secondary structure of RNA. and SpliceAid2 is a web resource for experimentally assessed splicing factors, RNA-binding sites. and branch point sequences.

## 3. Results

The analysis of medical and general data of 154 IS subjects (<50 years) and controls showed no difference regarding the age, sex, SBP, and DBP parameters, but the frequency of smoking was higher in IS patients (Table 1).

The rs10887800 AG frequency was greater in the IS group compared to controls (57.1 vs. 45.5 percent), but the difference was marginally nonsignificant (*P* = 0.054). Indeed, rs10887800 polymorphism was not accompanied with IS susceptibility in dominant (*P* = 0.122), recessive (*P* = 0.363), and allelic (*P* = 0.632) models. However, this variant was associated with IS in the overdominant model, and the AG genotype could lead to a 1.6-fold increased risk of IS compared to AA+ GG genotypes (OR = 1.6, 95% CI: 1-2.5, *P* = 0.033) (Table 2). No relationship was observed between *RNLS* rs2576178 polymorphism and IS in all genetic models.

The analysis of the combination effect of rs10887800 and rs2576178 variants on IS susceptibility showed no significant association (Table 3). In addition, there was no relationship between rs10887800 and rs2576178 haplotypes and IS (Table 4). The results of the LD analysis showed the *D*′ = 0.26 and *r*^2^ = 0.016 for rs10887800 and rs2576178 variants.

**Figure 1:**
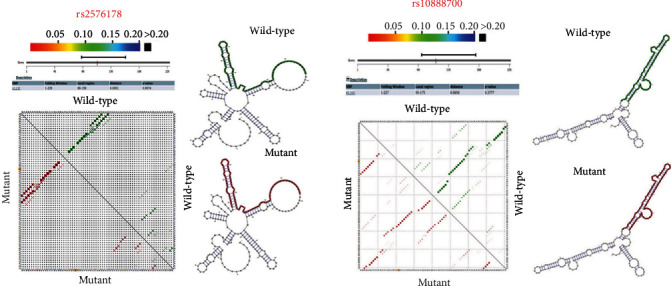
The effects of SNPs on local mRNA secondary structure.

**Figure 2:**
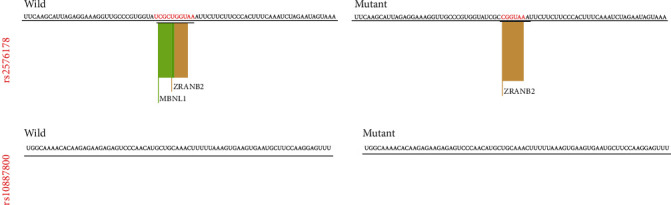
The SNPs effects on enhancers and silencers motifs.

The prediction of structural changes is resulted from rs2576178 and rs10887800 polymorphisms by RNAsnp that showed no alteration in the RNA structure (Figure 1). The SpliceAid2 analysis for rs2576178 showed that the alteration of RNA sequence “UCGCUGGUAA” to “UCGCCGGUAA” results in the lack of a MBNL1 protein binding site (Figure 2). The MBNL1 protein can act as an activator or repressor of splicing on different pre-mRNAs [20]. MBNLs are dsRNA binding factors that can bind CUG or CCUG repeats [21] and can modulate the selection of alternative splice sites in splicing of the *RNLS* gene. The analysis by spliceAid2 for rs10887800 SpliceAid2 did not show any protein binding site, and this SNP did not affect the binding factors.

## 4. Discussion

In this study, we evaluated the effects of *RNLS* rs10887800 and rs2576178 variants and IS on subjects < 50 years in Southeast Iran. We indicated no significant association between *rs2576178* polymorphism and IS in all models, but *RNLS* rs10887800 variant was associated with increased IS risk only in the overdominant model (AG vs AA+ GG).

Evidences suggest that renalase is closely related to blood pressure control [12]. Animal and clinical findings have proven the effect of the reduced renalase expression on elevated blood pressure [22]. Studies confirmed that renalase deficiency may lead to increased plasma catecholamine levels and subsequently increased blood pressure. In addition, the impact of renalase deficiency on declined tolerance to ischemia and ischemic myocardial impairment has been demonstrated in animal models. Regarding the roles of renalase, it is hypothesized that this enzyme can lead to hypertension and related disease [23]. Recently evidence introduces renalase as a signaling molecule which is similar to a cytokine that interacts with plasma membrane receptor to trigger protein kinase B and the mitogen-activated protein kinase pathway activation. Therefore, this enzyme can mediate a cytoprotective action in several diseases such as ischemic stroke [10].

Hypertension is presented as one of the most important risk factors of cardiovascular and cerebrovascular diseases [14]. The role of renalase in protection against hypertension, ischemic myocardial damage, and ischemic stroke has been confirmed in numerous animals' studies [22]. These findings suggest that renalase may play a protective role against ischemic injury, and recombinant renalase can be used in the inhibition and treatment of ischemic diseases [9]. The mechanism of the effect of renalase is not clea;, however, it is assumed that it may act inthe protection process against ischemic diseases by the regulation of cell necrosis, apoptosis, and local inflammatory reactions [24]. Lower activity of renalase in patients with a history of stroke or hypertension has been shown [15]. In addition, renalase has been described as a multifunctional enzyme which may play a key role in oxidative stress conditions [25].

Regarding the effect of genetic factors in addition to environmental causes in hypertension and IS etiology, numerous studies have investigated the impact of genetic variants on the renalase gene and several diseases like vascular and hypertensive complications [16, 17, 26].

Zhang et al. (2013) indicated a significant effect of rs10887800 and rs2576178 variants on ischemic stroke in patients with hypertension. Moreover, they found an association between rs2296545 and ischemic stroke in hypertension subgroups [16]. In their study, Li et al. (2014) found no relation between *RNLS* rs2576178, rs2296545, and rs10887800 variants and IS (mean age, 57.82 ± 9.92), but the rs10887800 GG genotype and G allele were associated with severe intracranial cerebral atherosclerotic vascular stenosis [17], which are consistent with our results regarding the role of the rs10887800 polymorphism in IS susceptibility in patients < 50 years.

The effect of *RNLS* polymorphisms on essential hypertension was examined by Zhao et al. (2007) and showed that the rs2576178 G and rs2296545 C alleles were more frequent in patients with this disease [26]. In contrast, Fava et al. (2012) refuted the effect of *RNLS* rs2576178 and rs2296545 variants on hypertension susceptibility [27]. The results of a meta-analysis from 4 studies performed by Shi et al. (2015) indicated that *RNLS* rs2296545 polymorphism is not related to hypertension in none of genetic models [28], but another meta-analysis by Lv et al. (2016) on six studies showed the relationship between rs2296545 but not rs2576178 variants and higher risk of hypertension [29]. The findings of a study conducted by Li el al. revealed higher frequency of rs2576178 A allele in hypertensive and coronary heart disease patients than hypertensive cases. Also, they showed the relationship between rs2296545 C allele and hypertension [30].

The findings of Buraczynska et al.'s study exhibited the rs2296545 C allele as a risk factor for hypertension in diabetic patients. Indeed, the rs2576178 alleles were different between hypertensive patients and controls, but not normotensive one. The rs10887800 G was differed in hypertensive patients with stroke as well [15]. Teimoori el al. indicated lack of relationship between rs10887800 and rs2576178 variants and PE, but they found that the GG/GG combined genotypes and G-G haplotype were associated with increased risk of this disorder. Indeed, they revealed the effect of rs10887800 variant on severe PE [19].

In addition, the results of in silico analysis showed no effect of rs2576178 and rs10887800 polymorphisms on the RNA structure. However, the A to G substitution of rs2576178 results in the loss of a MBNL1 protein binding site in the RNA structure. Indeed, RNLS rs10887800 showed no effect on the protein binding sites. Although, the findings of this insilico analysis are not consistent with our finding about the effect of rs10887800 but not rs2576178 polymorphism on IS susceptibility, and it is in line with results some studies. Therefore, the functional studies are necessary for approval of this bioinfirmatic analysis in experimental models.

In addition, as discussed above, the effect of *RNLS* polymorphisms on different diseases and various countries and ethnic groups was inconsistent; therefore, more researches in different regions and ethnicities are necessary to examine the impact of these variants on IS and hypertensive diseases. Moreover, if the assay of renalase activity can be accompanied with genetic evaluations, the results become more appreciated.

In conclusion, the current study showed the association of RNLS rs10887800 polymorphism and IS in the overdominant model (AG vs AA+ GG), but there was no relationship between RNLS rs2576178 polymorphism and IS in all genetic models. The in silico analysis showed that the rs2576178 polymorphism results in the lack of a MBNL1 protein binding site in RNA sequence.

## Figures and Tables

**Table 1:** Demographic characteristics of ischemic stroke patients and control group.

Variable	Controls, *n* = 165	IS, *n* = 154	*P* value
Age (years)	37.2 ± 10.8	36.8 ± 10.6	0.7^a^
Sex (male/female)	69/96	69/85	0.7^b^
Smoking (*n*, %)	29 (17.6)	43 (27.9)	0.03^b^
SBP (mmHg)	117 ± 18	119 ± 25	0.4^a^
DBP (mmHg)	75 ± 8.4	78 ± 18	0.1^a^

^a^Independent *t*-test. The data represent mean ± standard deviation (SD). ^b^Fisher exact test.

**Table 2:** Allelic and genotypic frequency of maternal *RNLS rs10887800* and *rs2576178* polymorphisms in IS patients and control group.

	IS (*N* = 154)	Control (*N* = 165)	*P* value	OR (95% CI)	
*RNLS rs10887800*					
Codominant					
AA, *n* (%)	41 (26.6)	57 (34.5)		1	
AG, *n* (%)	88 (57.1)	75 (45.5)	0.054	1.7 (1-2.7)	0.058
GG, *n* (%)	25 (33)	33 (20)	0.894	1.1 (0.5-2)	0.878
Dominant (AG + GG vs. AA)			0.122	1.5 (0.9-2.4)	0.126
Recessive (GG vs. AG + AA)			0.363	0.8 (0.4-1.4)	0.385
Overdominant (AG vs. AA+ GG)			0.033	1.6 (1-2.5)	0.037
Allele					
A, *n* (%)	170 (55)	189 (57)		1	
G, *n* (%)	138 (45)	141 (43)	0.632	1.1 (0.8-1.5)	
*RNLS rs2576178*					
AA, *n* (%)	115 (74.7)	115 (69.7)		1	
AG, *n* (%)	32 (20.8)	42 (25.5)	0.306	0.8 (0.5-1.3)	0.313
GG, *n* (%)	7 (4.5)	8 (4.8)	0.756	0.9 (0.3-2.4)	0.803
Dominant model (AG + GG vs. AA)			0.307	0.8 (0.5-1.3)	0.323
Recessive model (GG vs. AG + AA)			0.855	0.9 (0.3-2.6)	0.899
Overdominant (AG vs. AA+GG)			0.321	0.8 (0.5-1.3)	0.324
*Allele*					
A, *n* (%)	262 (85)	272 (82)		1	
G, *n* (%)	46 (15)	58 (18)	0.392	0.8 (0.5-1.3)	

**Table 3:** The combination effects of *RNLS rs10887800* and *rs2576178* polymorphisms on IS risk.

*rs10887800*	*rs2576178*	IS (*N* = 154)	Control (*N* = 165)	*P* value	OR (95% CI)
AA	AA	35 (22.7)	41 (24.8)		1
AA	AG	6 (3.9)	15 (9.1)	0.16	0.5 (0.2-1.3)
AA	GG	0 (0)	1 (0.5)	—	—
AG	AA	64 (41.6)	56 (33.9)	0.3	1.3 (0.8-2.4)
AG	AG	20 (13)	14 (8.5)	0.2	1.7 (0.7-3.8)
AG	GG	4 (2.6)	5 (3)	0.9	0.9 (0.2-3.8)
GG	AA	16 (10.4)	18 (10.9)	0.9	1 (0.5-2.3)
GG	AG	6 (3.9)	13 (7.9)	0.3	0.5 (0.2-1.6)
GG	GG	3 (1.9)	2 (1.2)	0.6	1.8 (0.3-11)

**Table 4:** The haplotype analysis of *RNLS rs10887800* and *rs2576178* polymorphisms on IS risk.

*rs10887800*	*rs2576178*	PE (%)	Control (%)	*P* value
A	A	49.8	49.2	0.88
G	A	35.3	33.2	0.59
G	G	9.6	9.5	0.99
A	G	5.4	8.1	0.18

## Data Availability

The data used to support the findings of this study are included within the article.
